# Abuse Potential and Neurotoxic Effects of the Synthetic Cannabinoid 4F‐ABUTINACA Self‐Administration in Adult Male Rats

**DOI:** 10.1111/adb.70177

**Published:** 2026-07-02

**Authors:** Baobao Shi, Huizhen Liu, Manqing Wu, Yuting Wang, Dan Fu, Lili Wen, Huifen Liu, Peng Xu, Wenhua Zhou, Haitao Wang, Miaojun Lai

**Affiliations:** ^1^ School of Psychology and Mental Health North China University of Science and Technology Tangshan Hebei Province China; ^2^ Department of Psychiatry Affiliated Kangning Hospital of Ningbo University Ningbo Zhejiang China; ^3^ Shanghai Mental Health Center Shanghai Jiao Tong University School of Medicine Shanghai China; ^4^ Zhejiang Key Laboratory of Drug Addiction and Brain Health Ningbo Zhejiang China; ^5^ Office of China National Narcotics Control Commission China Pharmaceutical University Joint Laboratory on Key Technologies of Narcotics Control Beijing China; ^6^ Hebei Key Laboratory of Mental Health and Brain Science Tangshan Key Laboratory of Mental Health and Cognitive Neuroscience Tangshan China

**Keywords:** 4F‐ABUTINACA, abuse potential, intravenous self‐administration, neurotoxic effects, synthetic cannabinoid

## Abstract

4F‐ABUTINACA, a fourth‐generation synthetic cannabinoid, has been identified in branded herbal smoking mixtures and e‐cigarettes seized in China. However, its potential for abuse and its corresponding neurotoxic effects remain poorly understood. In the present study, we evaluated the abuse potential of 4F‐ABUTINACA using the intravenous self‐administration (IVSA) model and assessed anxiety‐like behaviour using the open‐field test (OFT) and the elevated plus maze test (EPM). Additionally, neuronal injury, apoptosis, alterations in glia expression and the BDNF‐TrkB‐AKT signalling pathway in multiple brain regions were assessed simultaneously. Rats acquired stable nose‐poke operant response for self‐administering 4F‐ABUTINACA (0.00625 mg·kg^−1^·infusion^−1^) and showed significant drug‐seeking behaviour induced by conditioned cues. Persistent anxiety‐like behaviours were observed both immediately after drug‐taking (SA group) and cue‐induced reinstatement testing after a 14‐day extinction period (CIR group). Histochemical analysis revealed more pronounced neuronal injury and apoptosis in the hippocampus, prefrontal cortex (PFC) and nucleus accumbens (NAc) in the SA group than in the CIR group. Reactive astrogliosis and microgliosis were observed in the hippocampus and the PFC in the SA group, whereas the numbers of microglia and astrocytes decreased in the NAc of the CIR group. We also found the distinct region‐specific alterations in the BDNF‐TrkB‐AKT signalling pathway expression profile between the SA and CIR groups. The present results demonstrate that 4F‐ABUTINACA exhibits significant potential for abuse and produces anxiety‐like behaviour during both the active drug‐taking and cue‐induced relapse stages, which are associated with the neuropathological alterations in specific brain regions.

## Graphical abstract

4F ABUTINACA maintains stable intravenous self‐administration at low doses in male rats, supporting its high abuse potential. Chronic exposure to 4F‐ABUTINACA induces persistent anxiety‐like behaviors and is associated with region‐specific neurotoxicity in the hippocampus, prefrontal cortex and nucleus accumbens, alongside dysregulation of the BDNF‐TrkB‐AKT signaling pathway.

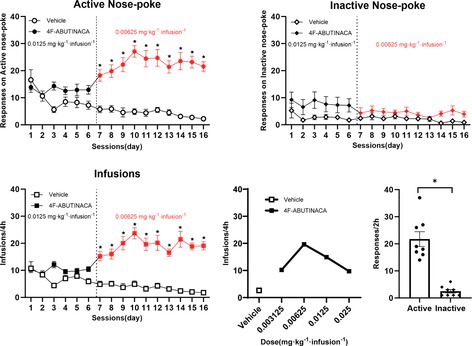

## 1 Introduction

The abuse of new psychoactive substances (NPS) has escalated significantly in recent years. According to a report by the United Nations Office on Drugs and Crime (UNODC), over 1200 NPS have been identified across 142 countries and regions by 2023, with synthetic cannabinoids (SCs) representing the largest within this group [1]. SCs have proliferated rapidly in the global illicit drug market due to their structural diversity, ease of synthesis, capacity to evade detection and circumvent regulation and accessibility. These factors pose a serious threat to public health worldwide.

Δ9‐tetrahydrocannabinol (THC), a partial agonist at cannabinoid receptors type 1 (CB1R) and type 2 (CB2R), is responsible for the addictive and reinforcing properties of cannabis as well as its observable effects on behaviour, appetite and nociception [2]. In rats [3], mice [4] and monkeys [5], the reinforcing effect of THC has been confirmed in the intravenous self‐administration (IVSA) model. SCs are structurally designed to mimic the effects of THC, acting as highly potent full agonists at CB1R and/or CB2R [6, 7], which confers elevated addiction liability and more severe toxicity profiles [8]. Accumulating evidence indicates that chronic or high‐dose exposure to SCs induces persistent cognitive deficits [9], mood disorders (e.g., anxiety [10] and depression [11]), neuropathological alterations [12] and in severe cases, fatal outcomes [13].

SCs are classified into four distinct generations based on structural evolution and pharmacological properties. The classic first‐generation SC, JWH‐018, binds to CB1R with a significantly higher affinity than THC [7]. 4F‐ABUTINACA (N‐(adamantan‐1‐yl)‐1‐(4‐fluorobutyl)‐1H‐indazole‐3‐carboxamide) is a fourth‐generation SC with an indazole amide core [14]. It was first detected in Asia in 2020 and has since been found in numerous branded herbal smoking mixtures and e‐cigarettes seized in China [15]. A fatal case has been reported involving 4F‐ABUTINACA combined with another SC, MDMB‐4en‐PINAC, highlighting the significant risks associated with its use [16]. Luo et al. found that 4F‐ABUTINACA, which exhibits a greater affinity for CB1R than JWH‐018, induces the typical ‘tetrad effect’ and produces biphasic, dose‐dependent effects in conditioned place preference (CPP) experiments in animal studies, with low doses inducing place preference and higher doses leading to place aversion [14]. However, no preclinical research has assessed the abuse potential of 4F‐ABUTINACA using the self‐administration model, which is the gold standard for evaluating the abuse liability.

The neurotoxic effects of SCs, including neuroinflammation, apoptosis and impaired neurogenesis, are primarily mediated through their interaction with CB1Rs, which are highly expressed in the central nervous system (CNS) [17]. Glial activation defends the CNS against neurotoxic insults and is characterized by rapid microglial phagocytic responses and the release of pro‐ and anti‐inflammatory mediators [18, 19]. Astrocytes, a type of glial cell, are increasingly recognized as important regulators of synaptic activity and plasticity [20]. They are therefore linked to the pathophysiology of neuropsychiatric conditions such as bipolar depression [21, 22], anxiety [23, 24] and drug addiction [25]. Exposure to THC or to SCs such as JWH‐018 leads to an increase in the expression of astrocytic markers (glial fibrillary acidic protein, GFAP) and microglial markers (ionized calcium‐binding adapter molecule 1, IBA‐1), suggesting glial activation [26, 27]. SCs have been implicated in the process of apoptosis mediated by oxidative stress in neuronal and glial cells, which leads to neurodegeneration [28]. In addition, previous studies have indicated an association between hippocampal neuroplasticity and mental disorders following SC exposure [29]. In particular, brain‐derived neurotrophic factor (BDNF), a critical regulator of neuroplasticity, has shown altered expression in animal models of substance abuse and schizophrenia, with similar findings in human studies [30]. Recent research has demonstrated that exposure to JWH‐018 in male mice resulted in a reduction of hippocampal BDNF levels [9, 31], which could be reversed by CB1R antagonist AM251 treatment [9]. Previous studies have demonstrated that 4F‐ABUTINACA impairs memory function and causes selective downregulation of stress‐response genes and mitochondrial‐related genes in adolescent mice [32]. Further research is required to ascertain the neurotoxic effects of 4F‐ABUTINACA and to identify the behavioural correlates.

We hypothesized that 4F‐ABUTINACA would maintain stable self‐administration, and that this voluntary drug intake would be associated with persistent anxiety‐like behaviours and specific neuropathological alterations in reward‐ and anxiety‐related brain circuits.

## 2 Materials and Methods

### 2.1 Animals

Adult male SPF‐grade Sprague–Dawley rats (290–320 g) were obtained from the Experimental Animal Center of Zhejiang Province in Hangzhou, China. Rats were housed in a temperature‐ and humidity‐controlled room, under a reversed 12‐h light/dark cycle, with temperature maintained at 21°C–24°C and humidity at about 55% ± 5%. Rats had free access to water and food in home cages. All animal studies were conducted in accordance with the Institutional Animal Care and Use Committee of Ningbo University.

### 2.2 Drugs

4F‐ABUTINACA was provided by the Drug Intelligence and Forensic Center, Ministry of Public Security, Beijing, China. The compound was dissolved in sterile saline containing 1.5% ethanol and 1.5% Tween‐80.

### 2.3 IVSA Experiments

Self‐administration training was conducted in operant conditioning chambers (30 × 30 × 30 cm^3^) (Anilab, China). Each chamber was equipped with two nose‐pokes, each with a yellow indicator light, and a central white ceiling light to provide visual cues. The rat was given drug infusions via a syringe pump (0.047 mL/s) connected to the rat through a stainless‐steel rotating joint and Tygon tubing.

#### 2.3.1 Surgical Procedures

Rats were anaesthetized with isoflurane and implanted with indwelling catheters in the right jugular vein. Postsurgical recovery lasted 5–7 days, during which catheters were flushed daily with saline (0.3 mL) containing penicillin (60 000 units) and heparin (20 units). As described previously, catheters were flushed and patency was checked during the training sessions [33].

#### 2.3.2 Drug Self‐Administration Training

Following surgical recovery, the rats were assigned to vehicle and 4F‐ABUTINACA groups for IVSA under a fixed‐ratio 1 (FR1) reinforcement schedule. Rats in the 4F‐ABUTINACA group underwent an initial 6‐day training phase at the dose of 0.0125 mg·kg^−1^·infusion^−1^. Subsequently, the dosage was reduced to 0.00625 mg·kg^−1^·infusion^−1^ in order to facilitate the acquisition of 4F‐ABUTINACA for at least 10 days, until the acquisition criteria were met over the last three consecutive sessions (variation in the mean infusion within ±20%, ≥ 80% of total responses directed to the active nose‐poke, with no upward or downward trend). The doses of 4F‐ABUTINACA for the self‐administration experiment and dose–response testing were selected based on the CB1 receptor affinity of 4F‐ABUTINACA compared with that of JWH‐018 [14], as well as the doses of JWH‐018 used in previous self‐administration studies [34]. Concurrently, rats in the vehicle group that were given saline containing 1.5% ethanol and 1.5% Tween‐80 underwent the same training as the 4F‐ABUTINACA group. During the daily 4‐h sessions, the illumination of a cue light within the active nose‐poke port signalled drug availability. A single active nose‐poke response triggered an infusion of 4F‐ABUTINACA or vehicle, accompanied by a 5‐s illumination of the house light and an audible activation of the infusion pump. Each infusion was followed by a 20‐s timeout period, during which active and inactive nose‐poke responses were recorded, but no programmed consequences occurred, with the cue light re‐illuminating at timeout termination. Inactive nose‐poke responses were also recorded, without any consequences. Following the successful establishment of stable self‐administration, an acquisition curve was generated. Throughout the training period, rats were provided ad libitum access to a daily ration of 20‐g standard chow during nontraining hours.

#### 2.3.3 Dose–Response Determination

After stable responding was acquired, subsequent dose substitution under the FR1 reinforcement schedule was performed to construct a full dose–response curve of 4F‐ABUTINACA (0.003125–0.025 mg·kg^−1^·infusion^−1^). Each dose was tested for three consecutive sessions, followed by three reinstatement sessions at the original training dose (0.00625 mg·kg^−1^·infusion^−1^) prior to introducing a new dose.

### 2.4 Behavioural Tests

The vehicle group underwent the open‐field test (OFT) and the elevated plus maze test (EPM) exclusively on the day following the final self‐administration session. Drug‐exposed rats were randomly assigned to two groups, which separately underwent behavioural assessments in OFT and EPM at two critical time points: immediately post‐IVSA of 4F‐ABUTINACA (SA group) and immediately after cue‐induced reinstatement testing (CIR group). This grouping scheme was applied consistently throughout the study.

#### 2.4.1 Cue‐Induced Reinstatement

Rats in the CIR group underwent an extinction period for 14 sessions during which the drug was not available. The cue‐induced reinstatement test was conducted after extinction training, where active nose‐pokes triggered the presentation of the cue (chamber light and pump sound) without drug delivery, whereas inactive nose‐pokes had no effect. The duration of the reinstatement session was 2 h.

#### 2.4.2 OFT

The OFT was performed according to established protocols, with minor adaptations [35]. The OFT was conducted in a chamber measuring 100 cm × 100 cm × 40 cm (length × width × height), with an infrared camera mounted directly overhead to monitor the animal's movements. Prior to each trial, the floor and walls of the arena were thoroughly cleaned with 75% alcohol to remove any potential environmental odours that could influence the animal's behaviour. Each animal was gently placed at the centre of the chamber and allowed to explore freely for a period of 10 min. The open field was divided into 25 equally spaced squares, with the central nine squares designated as the central zone.

#### 2.4.3 EPM

EPM testing was conducted according to previously published protocols, with minor adjustments [36]. The apparatus (Beijing Zhongshi Science and Technology, China) consisted of two opposing open arms (50 cm long, 10 cm wide) and two opposing enclosed arms (50 cm long, 10 cm wide, 30 cm high), forming a plus‐shaped configuration. The arms converge at a central square area (10 cm × 10 cm), and the entire apparatus was elevated 70 cm above the ground. After a 30‐min acclimation period in the laboratory, each rat was individually placed at the centre of the platform, facing one of the open arms, and allowed to explore the maze freely for 10 min. Each animal's movements were recorded using an overhead camera. To eliminate any potential olfactory cues, the maze was cleaned with 75% alcohol between trials.

### 2.5 Brain Tissue Preparation

Following the completion of behavioural assays, the rats were deeply anaesthetized using isoflurane. Post‐anaesthesia, the brains were rapidly excised, and the hippocampus (HIP), prefrontal cortex (PFC) and nucleus accumbens (NAc) were carefully isolated. These tissues were immediately frozen in liquid nitrogen and stored at −80°C for subsequent use. For histological staining experiments, after anaesthesia, the thoracic cavity of the rats was exposed, and approximately 250 mL of 0.9% sodium chloride solution was perfused via the heart to remove the blood. This was followed by 200 mL of 4% paraformaldehyde (PFA) fixative (Biosharp Life Science, BL539A). Once cardiac perfusion was completed, the brains were removed and immersed in 4% PFA overnight at 4°C for fixation. Subsequently, for haematoxylin–eosin (HE) staining, Nissl staining and TUNEL staining, the brain tissues underwent a graded dehydration series, were cleared in xylene and then embedded in paraffin. The embedded tissues were then sectioned for further analysis. For immunofluorescence, after overnight fixation in PFA at 4°C, the dissected brain specimens were cryoprotected by a graded dehydration process using a 30% sucrose solution at 4°C for 4–5 days, until the tissues sank to the bottom of the container. The sucrose‐infused tissues were then rapidly frozen and coronally sectioned into 20‐μm‐thick slices using a Leica CM1860 cryostat (Leica Microsystems, Germany) maintained at −25°C. For the neurochemical and histochemical analyses, a subset of animals was randomly selected from those that had completed the entire behavioural protocol, met all predefined inclusion criteria, maintained patent intravenous catheters and provided tissue of adequate quality for subsequent assays. All image acquisition and quantitative analyses were performed with the experimenter blinded to experimental group allocation.

### 2.6 Immunofluorescence

Immunofluorescence experiments were performed according to published protocols with minor modifications [35]. After three 5‐min rinses in phosphate‐buffered saline (PBS; pH 7.4, Servicebio, G4202) at room temperature (RT), free‐floating brain sections were permeabilized and blocked in PBS containing 0.3% Triton X‐100 (Solarbio, T8200) and 5% normal bovine serum albumin (BSA, Absin, abs9157) for 1 h at RT. The sections were then incubated with primary antibodies (anti‐Neun [Cell Signaling Technology, 94 403], 1:400; anti‐GFAP [Affinity Biosciences, DF6040], 1:800; anti‐IBA‐1 [Abcam, ab178846], 1:600) diluted in blocking buffer at 4°C for 12–16 h. After three 5‐min PBS washes at RT, slices were incubated with species‐matched secondary antibodies conjugated to fluorophores: Alexa Fluor 594 goat anti‐rabbit IgG (H + L) (Thermo Fisher Scientific, AB‐2534079), 1:500; Alexa Fluor 488 goat anti‐mouse IgG (H + L) (Thermo Fisher Scientific, AB‐2534069), 1:500 for 1 h in the dark. The sections were washed three times for 5 min with PBS. Finally, the sections were mounted using Mounting Medium With DAPI‐Aqueous, Fluorshield (Abcam, ab104139) and allowed to cure overnight before imaging.

### 2.7 Nissl Staining

As described previously [37], paraffin‐embedded tissue sections (4 μm thick) were dehydrated and stained with 0.5% Toluidine Blue (Servicebio, G1128) for Nissl stain for 5 min at RT. Differentiation was performed by briefly immersing the sections in glacial acetic acid (SCRC, 10000218), followed by dehydration through ascending ethanol gradients and clearing with xylene. Finally, the tissue sections were mounted permanently using Mounting Medium for Microscopy (Absin, abs9177).

### 2.8 HE Staining

Paraffin sections were deparaffinized, rehydrated and stained with HE according to the procedure described in Reference [37]. Following HE staining, sections were dehydrated through an ascending ethanol series, cleared in xylene and permanently mounted using Mounting Medium.

### 2.9 TUNEL Staining

Neuronal apoptosis was assessed using a combined approach of the Terminal deoxynucleotidyl transferase dUTP nick‐end labelling (TUNEL) assay and double immunofluorescent labelling (DIFL). Briefly, brain sections were co‐incubated overnight with the TUNEL reaction reagent (from the Cell Apoptosis Detection Kit, Beyotime, #C1090) and the anti‐NeuN primary antibody (Cell Signaling Technology, 94403, 1:400). The following day, sections were incubated with the Alexa Fluor‐conjugated secondary antibodies: Alexa Fluor 488 goat anti‐mouse IgG (H + L) (Thermo Fisher Scientific, AB‐2534069), 1:500.

### 2.10 Western Blot Analysis

Western blotting was conducted following previously established protocols [38], with minor modifications. Briefly, tissue samples were retrieved from storage at −80°C and thawed on ice for 10 min. The samples were then lysed in lysis buffer and centrifuged at 12 000 rpm for 20 min at 4°C to collect the supernatant. Protein concentrations were quantified, and equal amounts of protein were separated using sodium dodecyl sulphate‐polyacrylamide gel electrophoresis (SDS‐PAGE). The proteins were subsequently transferred onto a polyvinylidene fluoride (PVDF) membrane. To block non‐specific binding, the membrane was incubated with a 5% BSA solution for 1 h at RT. The membrane was then incubated overnight at 4°C with primary antibodies, including BDNF (1:1000, Abcam, ab108319), β‐actin (1:10000, ABclonal, AC026), TrkB (1:1000, Abcam, ab187041), AKT (1:1000, Cell Signaling Technology, 4685s) and Phospho‐AKT (1:1000, Cell Signaling Technology, 4060s). Following primary antibody incubation, the membrane was washed with tris‐buffered saline containing 0.1% Tween‐20 (TBST) for 15 min and then incubated with a secondary antibody (Goat Anti‐Rabbit IgG (H + L) HRP; 1:5000, Affinity Biosciences, S0001) at RT for 1 h. Protein bands were visualized using chemiluminescent detection, and band intensities were quantified with ImageJ software.

### 2.11 Data Analysis

All numerical data were statistically processed and expressed as mean ± standard error of the mean (SEM) using GraphPad Prism 9.0 software. Statistical significance was determined using a threshold of *p* < 0.05.

Data on self‐administration acquisition were recorded, including the number of active and inactive nose‐poke responses, as well as the number of infusions, during the 16‐day sessions. For analysis of self‐administration behaviour, a two‐way ANOVA was conducted to compare the responses per session between the 4F‐ABUTINACA and vehicle groups, followed by Sidak's multiple comparisons test. In the cue‐induced reinstatement test, Student's *t*‐test was employed to compare differences between active and inactive responses within the CIR group. Data from behavioural assessments, immunofluorescence and western blot experiments were analysed using one‐way ANOVA, with post hoc comparisons performed using Tukey's multiple comparisons test to evaluate differences between groups.

## 3 Results

### 3.1 IVSA of 4F‐ABUTINACA

**FIGURE 1:**
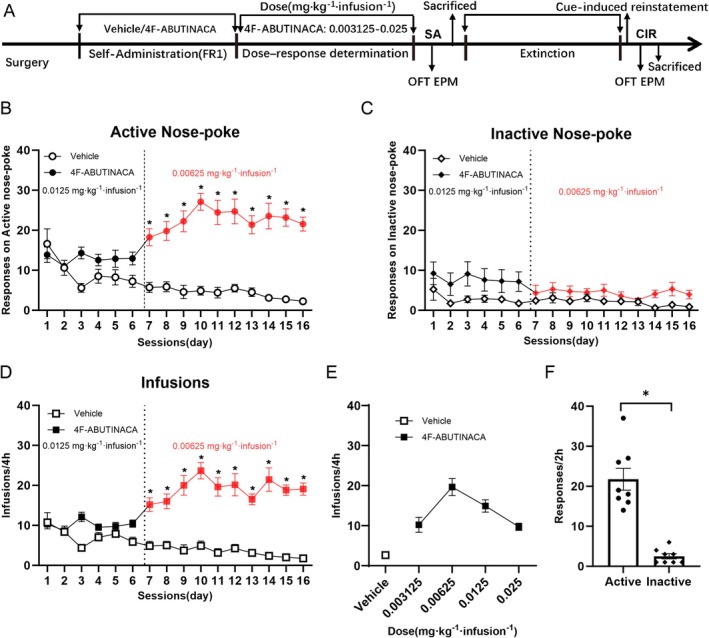
Self‐administration training of vehicle and 4F‐ABUTINACA. (A) Schematic diagram of the experimental setup. A comparative analysis was conducted between the vehicle (*n* = 8) and 4F‐ABUTINACA (*n* = 16) groups during the self‐administration phase, assessing (B) active response frequency, (C) inactive response frequency and (D) infusion counts. (E) Dose–response curve for intravenous self‐administration (IVSA) of 4F‐ABUTINACA. (F) Active and inactive responses during cue‐induced reinstatement in the CIR group (*n* = 8). Data are expressed as mean ± SEM. *p < 0.05 vs. vehicle group.

Three subjects in the 4F‐ABUTINACA group and one subject in the vehicle group did not complete the training protocol due to nonfunctional catheters and were thus excluded from analyses. All the other animals met the preset acquisition criteria. As shown in Figure 1B–D, two‐way ANOVA of the 4F‐ABUTINACA group and vehicle group during the self‐administration phase revealed distinct behavioural profiles. The 4F‐ABUTINACA group exhibited a significant increase in the number of active nose‐poke responses (*F*(1, 345) = 19.26, *p* < 0.0001, Figure 1B) and infusions (*F*(1, 345) = 265.2, *p* < 0.0001, Figure 1D) following a dose reduction to 0.00625 mg·kg^−1^·infusion^−1^, indicating that this group readily acquired 4F‐ABUTINACA self‐administration under the FR1 schedule by the end of the training session. Two‐way ANOVA revealed a main effect of response (*F*(1, 345) = 228.3, *p* < 0.0001) and a response × session interaction (*F*(15, 345) = 5.908, *p* < 0.0001). Sidak's post hoc test revealed significant differences in active nose‐poke responses between the vehicle group and the 4F‐ABUTINACA group across multiple sessions, with the 4F‐ABUTINACA group showing consistently higher responses in the 7th to 16th sessions (*p* values ranging from 0.0001 to 0.0004, Figure 1B). In the number of infusions, significant differences were revealed by Sidak's post hoc between the two groups in multiple sessions, particularly in sessions 7–16 (*p* < 0.0001 for most of these, Figure 1D). No significant intergroup differences were observed in the number of inactive nose‐poke responses (Figure 1C). Dose–response characterization (Figure 1E) displayed an inverted U‐shaped curve with peak efficacy at 0.00625 mg·kg^−1^·infusion^−1^, indicating that 4F‐ABUTINACA produced a bell‐shaped dose‐dependent effect. These findings validated that 4F‐ABUTINACA served as a reinforcer in rats. Student's *t*‐test revealed that the CIR group exhibited significantly higher active nose‐poke responses when exposed to the conditioned cue (*t* (14) = 6.871, *p* < 0.0001; Figure 1F), and the proportion of effective nose‐pokes was 89.18%. This confirms the successful reinstatement of drug‐seeking behaviour triggered by the cue.

### 3.2 Persistent Neuropsychiatric Phenotypes Post 4F‐ABUTINACA Administration

#### 3.2.1 OFT

**FIGURE 2:**
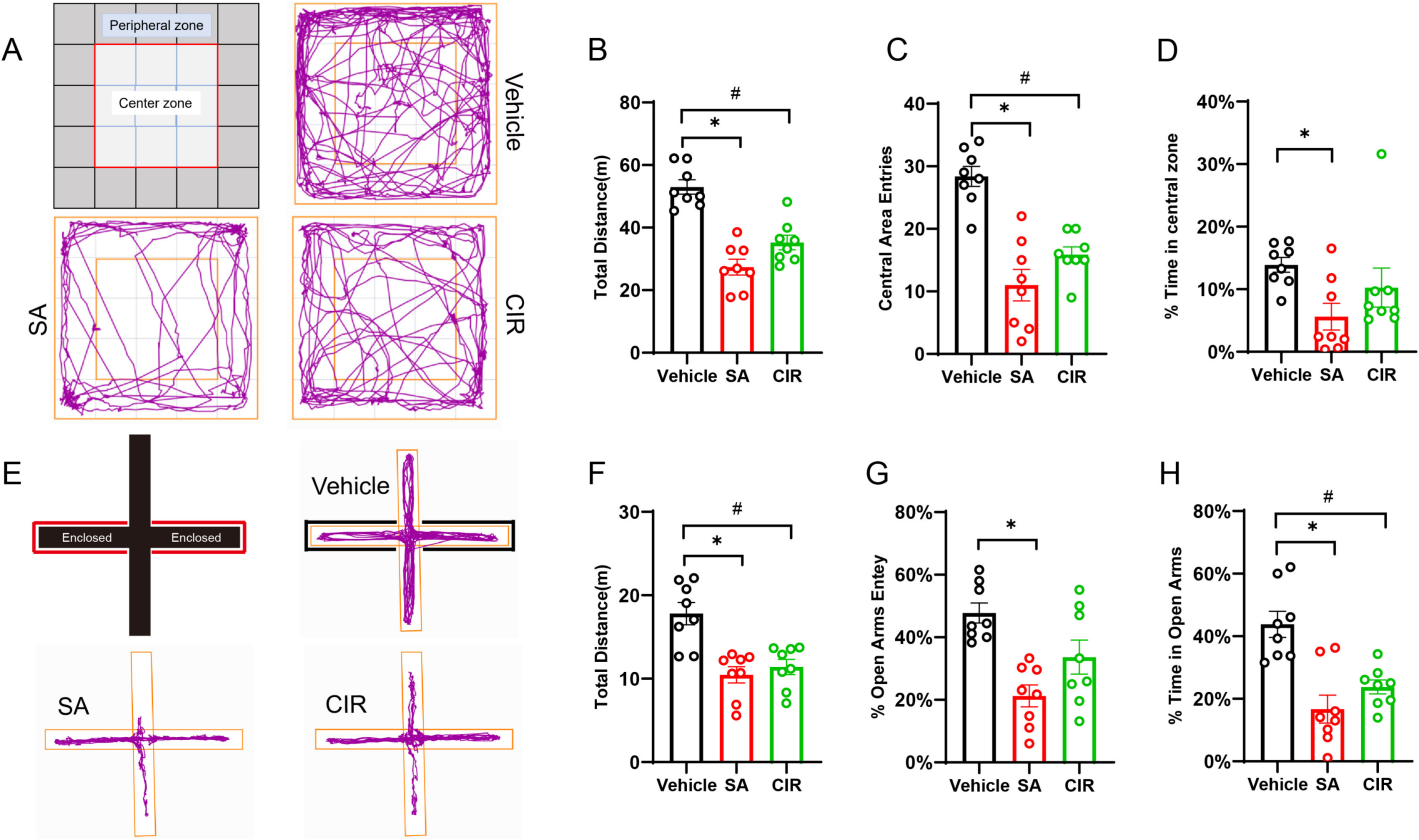
Results of behavioural tests performed on subjects treated with vehicle (*n* = 8) or 4F‐ABUTINACA (SA group, *n* = 8; CIR group, *n* = 8). (A) Representative locomotor trajectory plot from the open‐field test (OFT). (B) Total distance travelled in the OFT. (C) Number of entries into the central zone. (D) Percentage of time spent in the central area relative to the total test duration. (E) Representative locomotor trajectory plot from the elevated plus maze (EPM). (F) Total distance travelled in the EPM. (G) Percentage of entries into the open arms relative to the total number of entries. (H) Percentage of time spent in the open arms relative to the total test duration. Data are expressed as mean ± SEM. *p < 0.05, SA vs. vehicle; ^#^ *p* < 0.05, CIR vs. vehicle.

Following the pharmacological treatment, the rats exhibited a notable increase in baseline anxiety‐related behaviours. In the OFT, both the SA and CIR groups showed a significant reduction in total distance compared to the vehicle group (*F*(2, 21) = 30.03, *p* < 0.0001; SA vs. vehicle: *p* < 0.0001; CIR vs. vehicle: *p* < 0.0001, SA vs. CIR: *p* = 0.0743; Figure 2B). This locomotor suppression was accompanied by a reduction in the frequency of entries into the central area (*F*(2, 21) = 23.15, *p* < 0.0001; SA vs. vehicle: *p* < 0.0001; CIR vs. vehicle: *p* = 0.0003; SA vs. CIR: *p* = 0.1779; Figure 2C). The percentage of time spent in the central area showed a marginal decrease in the SA group compared to the vehicle group (*F*(2, 21) = 3.313, *p* = 0.0562; SA vs. vehicle: *p* = 0.0454; Figure 2D), but no statistically significant difference was observed in the CIR group (CIR vs. vehicle: *p* = 0.5085; CIR vs. SA: *p* = 0.3391). These results demonstrate that 4F‐ABUTINACA self‐administration induces persistent anxiety‐like behaviours in male rats.

#### 3.2.2 EPM

In the EPM test, both the SA and CIR groups showed a significant reduction in total distance travelled compared to the vehicle group (*F*(2, 21) = 13.38, *p* = 0.0002; SA vs. vehicle: *p* = 0.0003; CIR vs. vehicle: *p* = 0.0013; SA vs. CIR: *p* = 0.8182; Figure 2F). Furthermore, pharmacological treatment significantly attenuated exploration of anxiety‐provoking open arms. The percentage of entries into the open arms was significantly lower in treated rats (*F*(2, 21) = 10.17, *p* = 0.0008; SA vs. vehicle: *p* = 0.0005; CIR vs. vehicle: *p* = 0.0639; SA vs. CIR: *p* = 0.1127, Figure 2G), and a parallel decrease was observed in the percentage of time spent in the open arms (*F*(2, 21) = 14.08, *p* = 0.0001; SA vs. vehicle: *p* = 0.0001; CIR vs. vehicle: *p* = 0.0030; SA vs. CIR: *p* = 0.3907, Figure 2H).

By integrating the outcomes from the OFT and EPM, our findings demonstrate that 4F‐ABUTINACA self‐administration leads to anxiogenic effects. These effects are characterized by the avoidance of anxiogenic environments (open arms/central zones) and a generalized suppression of locomotor activity.

### 3.3 Effects of 4F‐ABUTINACA Self‐Administration on Neuronal Injury

To assess whether exposure to 4F‐ABUTINACA induces neuronal alterations, HE and Nissl staining were performed to examine morphological changes in neurons across hippocampal subregions (dentate gyrus [DG], CA1 and CA3), the PFC and the NAc in adult rats following IVSA. Furthermore, neuronal apoptosis was evaluated using a combined TUNEL/DIFL approach.

#### 3.3.1 HE Staining

**FIGURE 3:**
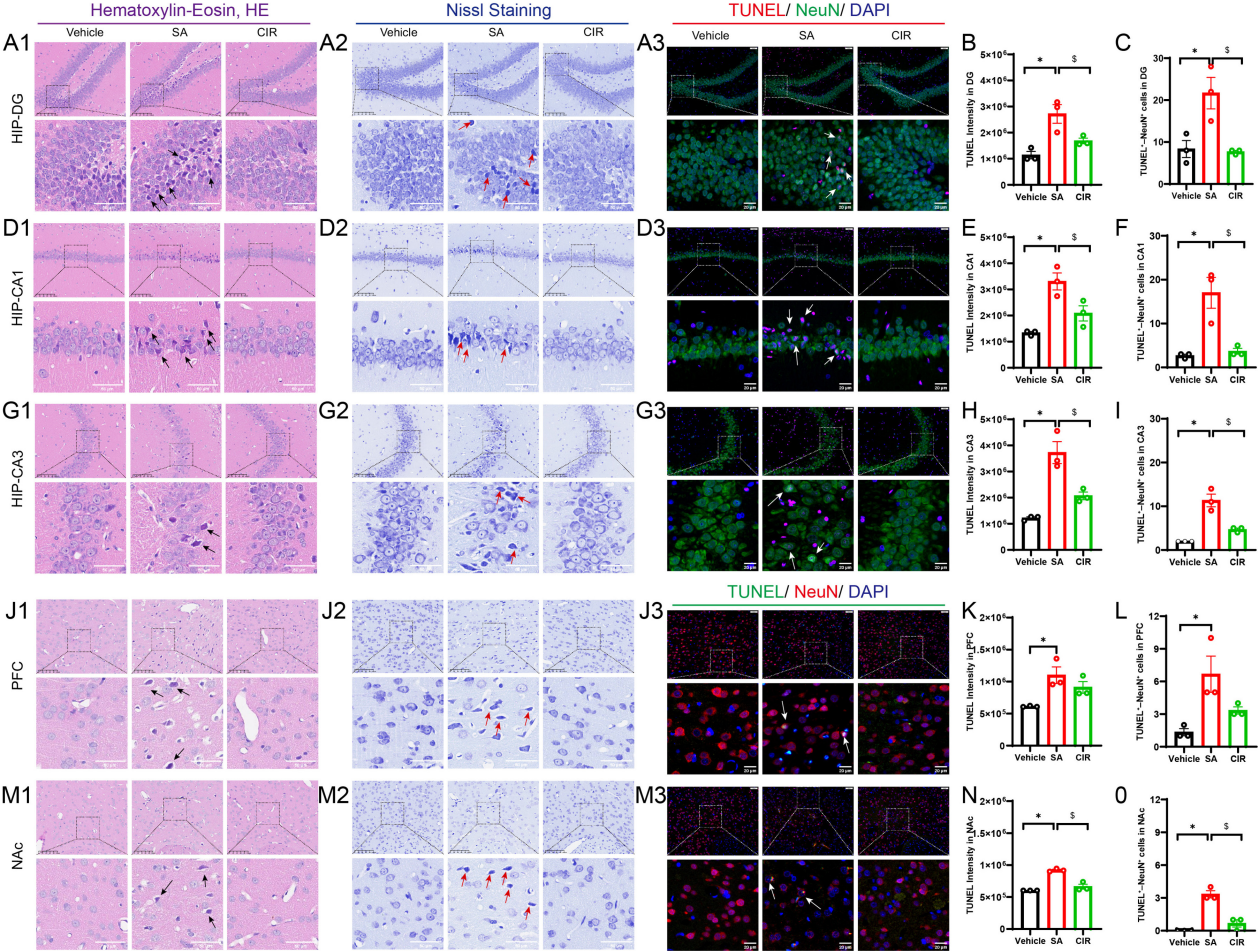
4F‐ABUTINACA self‐administration induced neuronal morphological changes and cell apoptosis. (A1, D1, G1, J1, M1) Representative micrographs of haematoxylin and eosin (HE) stained sections of the DG, CA1, CA3, PFC and NAc. The black arrows indicate damaged neurons. (A2, D2, G2, J2, M2) Representative micrographs of Nissl‐stained of the DG, CA1, CA3, PFC and NAc. The red arrows indicate neurons with impaired Nissl bodies. (A3, D3, G3, J3, M3) Representative micrographs of TUNEL/NeuN double‐stained sections of the DG, CA1, CA3, PFC and NAc. The white arrows indicate the apoptotic neurons. TUNEL (green in DG, CA1, CA3; red in PFC, NAc) was used to label apoptotic cells, NeuN (red in DG, CA1, CA3; green in PFC, NAc) was used to label neurons and DAPI (blue) was used to label the cell nuclei. (B, E, H, K, N) Quantification analysis of TUNEL fluorescence intensity in the DG, CA1, CA3, PFC and NAc. (C, F, I, L, O) Quantification of TUNEL/NeuN‐positive cells in the DG, CA1, CA3, PFC and NAc. Data are expressed as mean ± SEM (*n* = 3). *p < 0.05, SA vs. vehicle; ^#^ *p* < 0.05, CIR vs. vehicle; ^$^ *p* < 0.05, SA vs. CIR.

In the histological assessment using HE staining (Figure 3A1,D1,G1,J1,M1), the vehicle group demonstrated preserved cytoarchitecture, characterized by plump, eosinophilic somata and uniformly dispersed chromatin, with neurons maintaining normal laminar organization without signs of nuclear condensation or cytoplasmic shrinkage. In stark contrast, the SA group exhibited significant neurodegeneration, including a reduction in granule cell density, nuclear hyperchromasia with pyknosis and perinuclear halos, and diminished cytoplasmic eosinophilia. The CIR group showed partial neuronal restoration, as evidenced by recovery in granule cell density, a reduced frequency of pyknotic nuclei and the restoration of cytoplasmic eosinophilia to levels approaching those observed in the vehicle group.

#### 3.3.2 Nissl Staining (Figure 3A2,D2,G2,J2,M2)

The SA group displayed a significant reduction in neuronal density across critical brain regions compared to both the vehicle and CIR groups. Upon closer examination, neurons in the SA group presented distinctive degenerative morphological features. These neurons exhibited hyperchromatic, shrunken somata accompanied by karyopyknosis and a notable loss of Nissl substance, indicative of early necrotic changes. In contrast, neurons from the CIR group preserved a cytoarchitecture nearly identical to that of normal controls, suggesting resilience to the degenerative processes observed in the SA group.

#### 3.3.3 TUNEL Staining

Quantitative analysis using one‐way ANOVA revealed a significant effect of group on TUNEL fluorescence intensity across all observed brain regions (DG: *F*(2, 6) = 12.08, *p* = 0.0079; CA1: *F*(2, 6) = 15.32, *p* = 0.0044; CA3: *F*(2, 6) = 24.54, *p* = 0.0013; PFC: *F*(2, 6) = 7.206, *p* = 0.0254; NAc: *F*(2, 6) = 52.23, *p* = 0.0002). Post hoc Tukey's multiple comparisons test showed that the fluorescence intensity was significantly higher in the SA group compared to the vehicle group (DG: *p* = 0.0069, Figure 3B; CA1: *p* = 0.0037, Figure 3E; CA3: *p* = 0.0011, Figure 3H; PFC: *p* = 0.0218, Figure 3K; NAc: p = 0.0002, Figure 3N), while it was markedly reduced in the CIR group compared to the SA group (DG: *p* = 0.0438; CA1: *p* = 0.0334; CA3: *p* = 0.0093; NAc: *p* = 0.0006), indicating elevated apoptosis in the SA group.

To specifically quantify neuronal apoptosis, we performed TUNEL/NeuN double staining. One‐way ANOVA revealed a significant effect of group on the density of TUNEL‐positive neurons across all brain regions examined (DG: *F*(2, 6) = 10.21, *p* = 0.0117; CA1: *F*(2, 6) = 14.91, *p* = 0.0047; CA3: *F*(2, 6) = 31.20, *p* = 0.0007; PFC: *F*(2, 6) = 7.259, *p* = 0.0250; NAc: *F*(2, 6) = 42.00, *p* = 0.0003). Post hoc analysis confirmed that the SA group exhibited a significantly higher density of TUNEL‐positive neurons compared to the vehicle group in the HIP (DG: *p* = 0.0206, Figure 3C; CA1: *p* = 0.0066, Figure 3F; CA3: *p* = 0.0006, Figure 3I), as well as in the PFC (*p* = 0.0217, Figure 3L) and NAc (*p* = 0.0003, Figure 3O). Conversely, the density of TUNEL‐positive neurons was significantly lower in the CIR group than in the SA group (DG: *p* = 0.0167; CA1: *p* = 0.0093; CA3: *p* = 0.0037; NAc: *p* = 0.0011).

### 3.4 4F‐ABUTINACA Induces Changes in IBA‐1 and GFAP Immunoreactivity in Specific Brain Areas

A growing body of evidence demonstrates that glial‐mediated neuroimmune responses play a pivotal role in the pathogenesis and persistence of addictive behaviours. To assess the effects of intravenous 4F‐ABUTINACA administration on microglial (IBA‐1) and astrocytic (GFAP) density across different brain regions, quantitative analyses were performed for each brain area separately.

**FIGURE 4:**
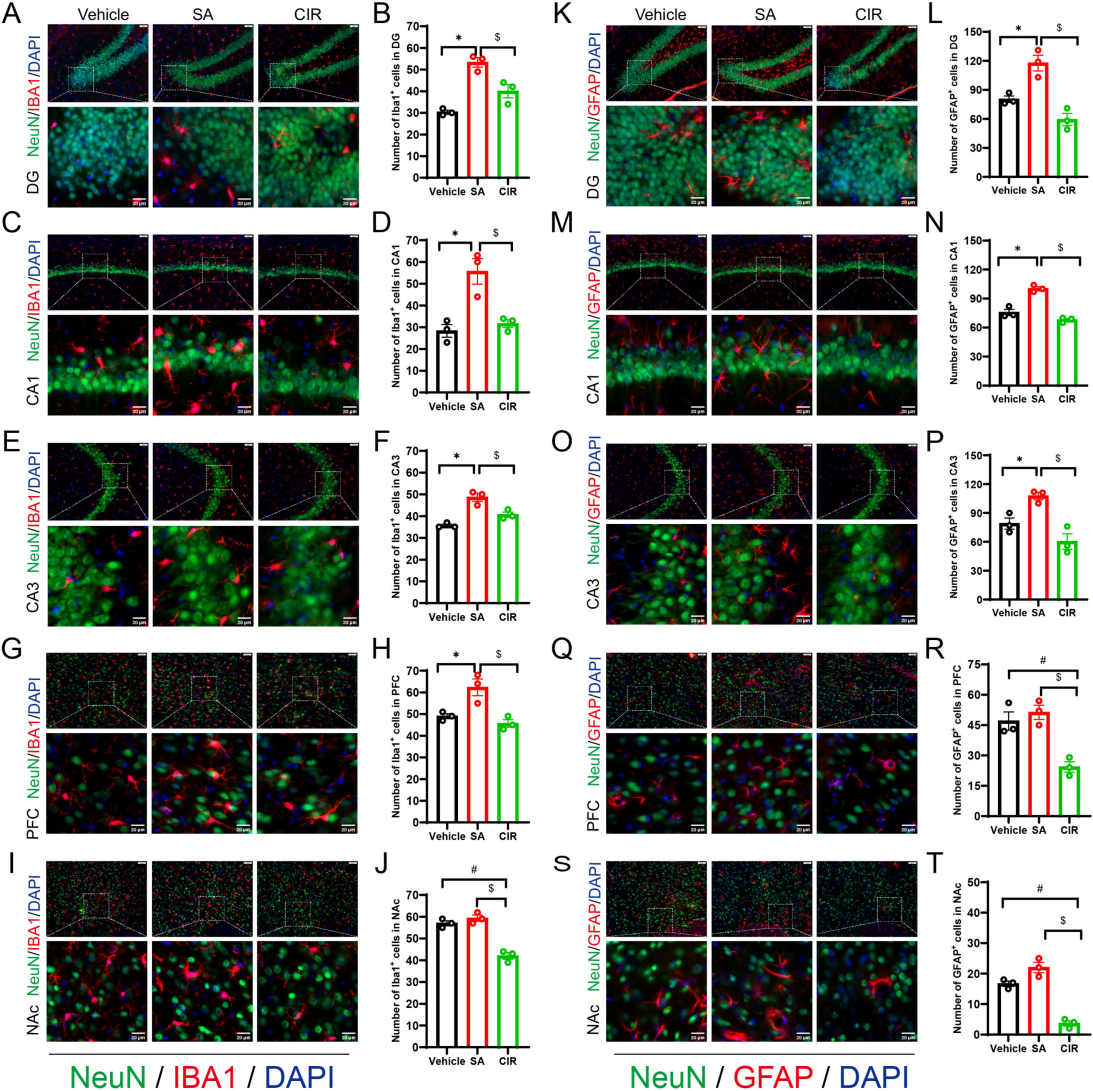
4F‐ABUTINACA self‐administration induced alterations in microglial (IBA‐1) and astrocytic (GFAP) immunoreactivity. A—J: Representative images of different brain regions obtained through fluorescence microscopy stained for IBA‐1 (red), NeuN (green, neuronal marker) and DAPI (blue, nuclear stain) in the three groups. The scale bar represents 20 μm. K–T: Representative images of different brain regions obtained through fluorescence microscopy stained for GFAP (red), NeuN (green) and DAPI (blue) in each group. The scale bar represents 20 μm. Quantitative data are expressed as mean ± SEM (*n* = 3). *p < 0.05, SA vs. vehicle; ^#^ *p* < 0.05, CIR vs. vehicle; ^$^ *p* < 0.05, SA vs. CIR.

Quantitative analysis using one‐way ANOVA followed by Tukey's multiple comparisons test revealed a significant increase in IBA‐1‐labelled microglial density in the DG (Figure 4B), CA1 (Figure 4D), CA3 (Figure 4F) and PFC (Figure 4H) of the SA group compared to the vehicle group (DG: *F*(2, 6) = 26.87, *p* = 0.0010, SA vs. vehicle: *p* = 0.0008; CA1: *F*(2, 6) = 14.30, *p* = 0.0052; SA vs. vehicle, *p* = 0.0065; CIR vs. vehicle, *p* = 0.8265; CA3: *F*(2, 6) = 24.19, *p* = 0.0013; SA vs. vehicle, *p* = 0.0011; PFC: *F*(2, 6) = 12.14, *p* = 0.0078; SA vs. vehicle, *p* = 0.0229). Compared to the SA group, microglial density was significantly lower in the CIR group across all examined regions, including the DG (*p* = 0.0130), CA1 (*p* = 0.0120), CA3 (*p* = 0.0128), PFC (*p* = 0.0083) and NAc (*p* = 0.0003, Figure 4J). In contrast, the NAc exhibited an opposite pattern: The CIR group exhibited a marked reduction in Imicroglial density compared to the vehicle group (*F*(2, 6) = 45.94, *p* = 0.0002, SA vs. vehicle, *p* = 0.5011; CIR vs. vehicle, *p* = 0.0006).

Parallel analysis revealed that the SA group exhibited a significant increase in astrocytic density in the DG (*F*(2, 6) = 22.19, *p* = 0.0017; SA vs. vehicle: *p* = 0.0133, Figure 4L), CA1 (*F*(2, 6) = 51.72, *p* = 0.0002; SA vs. vehicle: *p* = 0.0008, Figure 4N), CA3 (*F*(2, 6) = 14.89, *p* = 0.0047; SA vs. vehicle: *p* = 0.0386, Figure 4P) compared to the vehicle group. Conversely, GFAP‐labelled astrocyte density was significantly lower in the CIR group than in the SA group across all examined regions, including the DG (*p* = 0.0014), CA1 (*p* = 0.0002), CA3 (*p* = 0.0039), PFC (*p* = 0.0045, Figure 4R) and NAc (*p* = 0.0001, Figure 4T). While astrocytic density was significantly reduced in the CIR group relative to the vehicle group in the PFC (*p* = 0.0105) and NAc (*p* = 0.0007), it failed to produce a similar reduction in the HIP (*p* > 005). These region‐specific alterations in glial density are summarized in Table 1.

**TABLE 1:** Expression of glial cells across different brain regions.

	DG			CA1			CA3			PFC			NAc		
	SA	CIR1	CIR2	SA	CIR1	CIR2	SA	CIR1	CIR2	SA	CIR1	CIR2	SA	CIR1	CIR2
IBA‐1	↑	ns	↓	↑	ns	↓	↑	ns	↓	↑	ns	↓	ns	↓	↓
GFAP	↑	ns	↓	↑	ns	↓	↑	ns	↓	ns	↓	↓	ns	↓	↓

*Note:* SA: comparison between SA group and vehicle group CIR1; comparison between CIR group and vehicle group CIR2; comparison between CIR group and SA group. Statistical symbols: ↑: significantly increased (*p* < 0.05); ↓: significantly decreased (*p* < 0.05); ns: not significant (*p* > 0.05).

### 3.5 4F‐ABUTINACA Alters BDNF Signalling Pathway in Specific Brain Regions

#### 3.5.1 Signalling Protein Expression in the HIP

**FIGURE 5:**
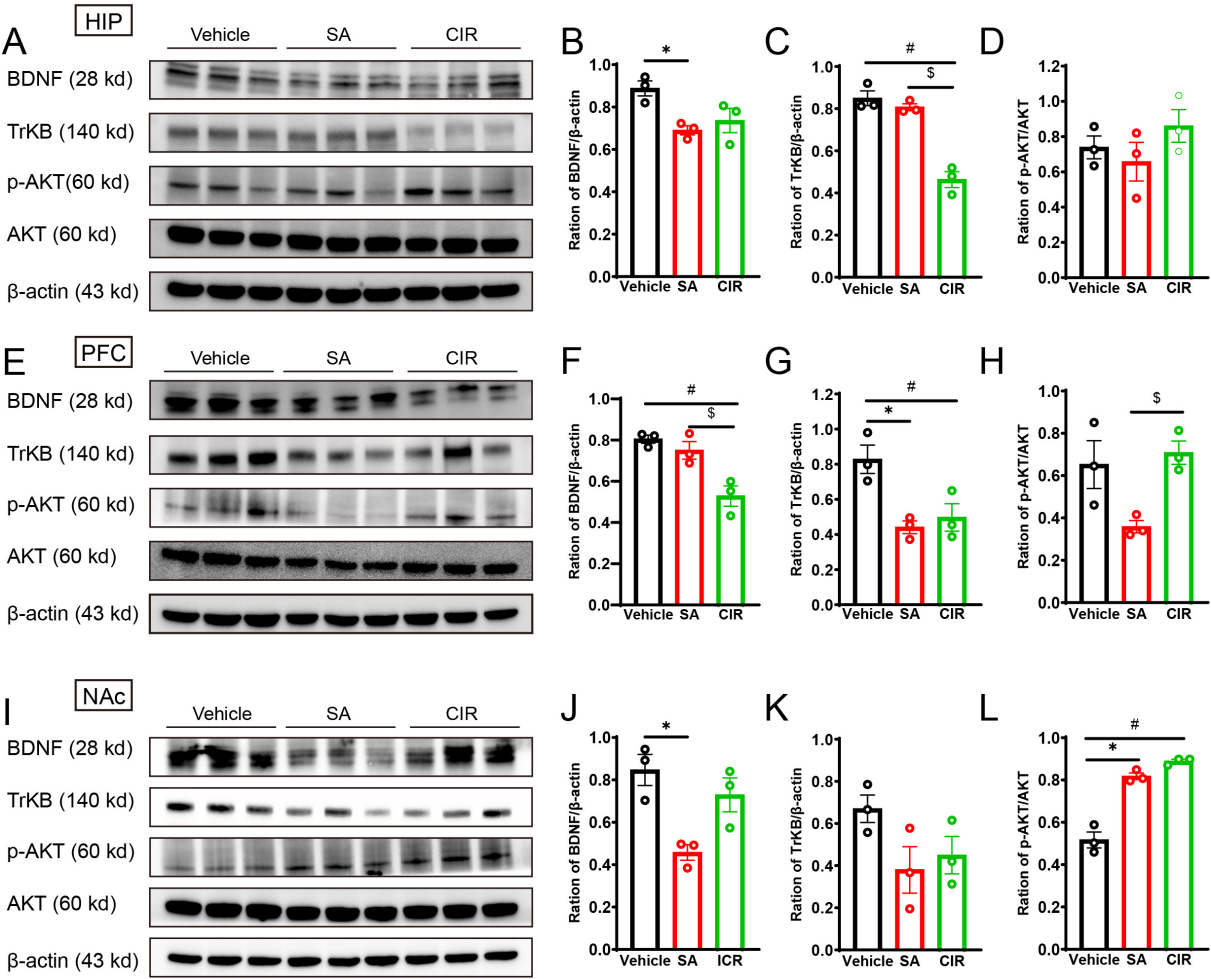
Effect of 4F‐ABUTINACA self‐administration on BDNF and its associated signalling pathway protein levels. Representative western blot images of BDNF, TrkB, p‐AKT, AKT and β‐actin in the HIP (A), PFC (E) and NAc (I). Quantified BDNF protein levels normalized to β‐actin in the HIP (B), PFC (F) and NAc (J). Quantified TrkB levels normalized to β‐actin in the HIP (C), PFC (G) and NAc (K). Quantified p‐AKT/AKT ratios normalized to total AKT in the HIP (D), PFC (H) and NAc (L). All quantitative data are presented as mean ± SEM (*n* = 3). *p < 0.05, SA vs. vehicle; ^#^ *p* < 0.05, CIR vs. vehicle; ^$^ *p* < 0.05, SA vs. CIR.

Significant downregulation of BDNF was observed in the SA group compared to the vehicle group, whereas no significant difference was observed between the CIR group and the vehicle group (*F*(2, 6) = 6.415, *p* = 0.0324; SA vs. vehicle, *p* = 0.0326; CIR vs. vehicle, *p* = 0.0864; Figure 5B). A marked reduction in TrkB expression was detected in the CIR group relative to the vehicle group and SA group (*F*(2, 6) = 45.90, *p* = 0.0002, CIR vs. vehicle: *p* = 0.0003; CIR vs. SA, *p* = 0.0006; Figure 5C). Neither the SA nor the CIR group differed significantly from the vehicle group in p‐AKT/AKT ratios (*F*(2, 6) = 1.268, *p* = 0.3473; SA vs. vehicle, *p* = 0.8069; CIR vs. vehicle, *p* = 0.6335; Figure 5D).

#### 3.5.2 Signalling Protein Expression in the PFC

Relative protein levels of BDNF, TrkB, AKT and p‐AKT in the PFC are shown in Figure 5E. Significant downregulation of BDNF was observed in the CIR group compared to both the vehicle group and SA group (*F*(2, 6) = 13.80, *p* = 0.0057, CIR vs. vehicle, *p* = 0.0061; CIR vs. SA, *p* = 0.0170; Figure 5F). Both the SA and CIR groups showed significant decreases in TrkB protein expression relative to the vehicle group (*F*(2, 6) = 9.357, *p* = 0.0143, SA vs. vehicle, *p* = 0.0167; CIR vs. vehicle, *p* = 0.0326; Figure 5G). AKT activation, assessed by the p‐AKT/AKT ratio, showed a nonsignificant downward trend in the SA group but partially recovered in the CIR group, with a significant increase compared to the SA group (*F*(2, 6) = 6.376, *p* = 0.0328, SA vs. vehicle, *p* = 0.0705; CIR vs. vehicle, *p* = 0.8590; CIR vs. SA, *p* = 0.0366; Figure 5H).

#### 3.5.3 Signalling Protein Expression in the NAc

Relative protein levels of BDNF, TrkB, AKT and p‐AKT in the NAc are shown in Figure 5I. One‐way ANOVA revealed a significant decrease in BDNF levels in the SA group compared to the vehicle group, although no significant difference was observed in the CIR group (*F*(2, 6) = 9.130, *p* = 0.0151, SA vs. vehicle, *p* = 0.0140; CIR vs. vehicle, *p* = 0.4674; Figure 5J). TrkB expression exhibited a nonsignificant downward shift in both the SA and CIR groups relative to the vehicle group (*F*(2, 6) = 2.816, *p* = 0.1372, SA vs. vehicle, *p* = 0.1363; CIR vs. vehicle, *p* = 0.2713; Figure 5K). While the p‐AKT/AKT ratio demonstrated significant increases (*F*(2, 6) = 65.71, *p* < 0.0001, SA vs. vehicle, *p* = 0.0003; CIR vs. vehicle, *p* < 0.0001; Figure 5L), indicating enhanced AKT pathway activation independent of TrkB‐mediated signalling during both acute processing and chronic phases.

## 4 Discussion

The present study demonstrated that 4F‐ABUTINACA maintains self‐administration under the FR1 schedule in male rats. Voluntary consumption of 4F‐ABUTINACA induces persistent anxiety‐like behaviours, with region‐specific neuronal injury and apoptosis, glial activation and the dysregulation of the BDNF‐TrkB‐AKT signalling pathway in the HIP, PFC and NAc. These findings highlight the potential risks associated with the use of 4F‐ABUTINACA.

The IVSA paradigm is commonly employed to evaluate the reinforcing effects of NPS in animal models [33]. Early studies documented the inability of THC to reliably maintain IVSA in rodents [39, 40]. However, more recent research has shown that THC readily maintains self‐administration at low doses [41]. The IVSA of SCs is not unprecedented, as several SCs have been shown to maintain self‐administration responding in rats [34, 42] and mice [43]. Our first critical finding is that rats developed stable drug‐seeking behaviour for 4F‐ABUTINACA under an FR1 schedule, specifically at a low dose of 0.00625 mg·kg^−1^·infusion^−1^, whereas no reliable drug‐taking behaviour emerged at 0.0125 mg·kg^−1^·infusion^−1^. Similarly, a previous study revealed that the SC PB‐22 supported IVSA at lower doses but failed to maintain responding at higher doses [44]; likewise, both JWH‐018 [34, 45] and WIN 55,212‐2 [46] have been shown to support reliable self‐administration under an FR1 schedule. In CPP experiments, a lower dose of 4F‐ABUTINACA significantly increased CPP scores; however, as the dose increased, CPP scores declined [14]. This phenomenon may be attributed to the typical ‘tetrad effect’ associated with elevated SCs concentrations, which likely induces aversive responses rather than rewarding effects [14, 47]. The neuropathological effects of SCs are highly complex, influenced by variables such as dose, exposure duration and sex. THC and related cannabinoids can elicit a range of emotional responses in both humans and experimental animals, including anxiolytic effects at low doses, whereas higher doses are more likely to cause anxiety‐like behaviour [48]. Adolescent male mice exposed to JWH‐018 exhibit anxiety‐like behaviours, whereas adult mice display the opposite effects. Interestingly, female mice demonstrate anxiety‐like behaviours following JWH‐018 exposure, whereas males do not [49]. In our study, adult rats that self‐administered 4F‐ABUTINACA exhibited significant anxiety‐like behaviour, similar to those observed in rats exposed to JWH‐018 [50]. However, Kai et al. found no significant changes in anxiety‐like behaviours after a single dose of 4F‐ABUTINACA (1, 2 and 4 mg/kg) administered to adolescent or adult mice [32]. In contrast, exposure to AB‐CHMINACA and PB‐22 has been shown to reduce anxiety levels [36]. These contrasting behavioural outcomes are likely dependent on the specific characteristics of compounds, dosing parameters and the duration or timing of exposure.

Acute or chronic exposure to addictive substances induces brain region‐specific glial responses [51, 52]. Increasing evidence points to neuroimmune dysregulation as a key pathological mechanism underlying anxiety disorders. Within the CNS, immune‐inflammatory responses activate both astrocytes and microglia, leading to the release of proinflammatory cytokines, which in turn cause neuronal damage. This process is strongly associated with anxiety‐like behaviours [53]. Exposure to SCs induces neuroinflammatory responses, which are related to neuronal cell apoptosis and microglial activation [54]. Our findings show that exposure to 4F‐ABUTINACA significantly reduced neuronal density, caused marked alterations in neuronal morphology and greatly increased neuronal apoptosis. These results align with previous studies, in which chronic exposure to AB‐FUBINACA leads to a reduction in hippocampal neuronal density [12]. Additionally, exposure to CUMYL‐4CN‐BINACA was also linked to neuronal apoptosis [55]. Another key finding of the study is that IVSA of 4F‐ABUTINACA led to sustained activation of microglia and astrocytes in the rat HIP. Consistent with our findings, Pintori et al. [50] found that exposure to JWH‐018 during adulthood induces persistent gliosis, characterized by reactive astrogliosis and microglial activation, with these changes lasting at least 7 days after drug cessation. In contrast, our findings show that the expression of glia in the HIP decreased following a 14‐day extinction period, compared to the immediate results observed after self‐administration of 4F‐ABUTINACA. The HIP plays a crucial role in the regulation of emotional behaviours, particularly anxiety states [56]. The hippocampal subregions—the DG, CA1 and CA3—are involved in the acquisition of contextual fear, contributing to anxiety [57]. Our findings suggest that the increased expression of microglia and astrocytes in the DG, CA1 and CA3 may underlie the anxiety‐like behaviours observed in rats self‐administering 4F‐ABUTINACA.

Glutamatergic dysregulation after chronic self‐administration of a number of drugs of abuse has been directly linked to disrupted astroglial mechanisms of glutamate regulation in the nucleus accumbens core (NAcore) [58]. Drugs of abuse promote cue reactivity through downregulation of glutamate transporter 1 on NAcore astrocytes, leading to reduced glutamate uptake capacity [59]. GFAP expression was significantly reduced in the NAcore following cocaine self‐administration and extinction [60]. Moreover, engaging NAcore astrocytes has been shown to inhibit the cued methamphetamine‐seeking behaviours [61]. In the present study, the expression of microglia and astrocytes in the NAc decreased following extinction, which may contribute to cue‐induced 4F‐ABUTINACA‐seeking behaviours. Overall, our findings suggest a link between 4F‐ABUTINACA exposure and changes in the expression of microglia and astrocytes in the adult rat brain.

The BDNF‐TrkB‐AKT signalling pathway is crucial for regulating neuronal survival, synaptic plasticity and cognitive processes. In substance use disorder research, this pathway plays a role in both the reinforcement and withdrawal stages of addiction to substances like cocaine and methamphetamine [62, 63]. Our study identified region‐specific reductions in BDNF expression, consistent with the findings that JWH‐018 and AB‐FUBINACA markedly reduced hippocampal BDNF levels in mice [12, 31]. In contrast, WIN 55,212‐2 increased BDNF expression in the rat PFC [64]. Chronic exposure to SCs has been extensively associated with cognitive impairments [31], and BDNF expression may underlie these cognitive deficits. Although the present study focused on anxiety‐like behaviour rather than learning and memory performance following 4F‐ABUTINACA exposure, our results suggest that the downregulation of BDNF may be closely implicated in both the anxiety‐like behaviours and cue‐induced seeking behaviours observed.

Although this study demonstrated the abuse potential of 4F‐ABUTINACA at low cost, a limitation is that behaviour under the higher FR schedule, such as FR3, was not evaluated, and neither progressive‐ratio schedule nor behavioural economic demand procedures were employed to quantify the reinforcing efficacy. Another limitation of the present study is its exclusive use of adult male rats, which precludes an investigation of potential sexually dimorphic behavioural responses. Considerable evidence indicates significant sex differences in cannabinoid effects. SCs can lead to sex‐specific long‐term alterations in anxiety‐like behaviours, cognitive function and sensorimotor gating in adulthood [49, 65]. These differences likely originate from the modulatory influence of gonadal hormones on the endocannabinoid system and its downstream signalling pathways [66, 67]. Consequently, future work should systematically compare the addictive behaviours, neurotoxicity and molecular mechanisms of 4F‐ABUTINACA in both sexes and examine the regulatory role of the estrous cycle. In future studies, combinatorial gain‐of‐function (e.g., BDNF overexpression) or loss‐of‐function strategies should be employed to dissect causal mechanisms. These systematic interventions would clarify the role of BDNF pathway modulation in 4F‐ABUTINACA‐induced drug‐seeking behaviours and anxiety‐like phenotypes, thus significantly enhancing the translational potential of these findings.

## Author Contributions

Wenhua Zhou, Miaojun Lai and Haitao Wang designed the experiment and interpreted the results. Baobao Shi and Miaojun Lai wrote the original draft. Manqing Wu, Huizhen Liu, Yuting Wang, Dan Fu, Lili Wen, Huifen Liu and Peng Xu reviewed and edited the manuscript.

## Funding

This work was supported by Ningbo Public Welfare Research Project (2024S025); Ningbo Top Medical and Health Research Program (No. 2022030410); National Key R&D Program of China (2022YFC3300905); and National Natural Science Foundation of China (82471516).

## Conflicts of Interest

The authors declare no conflicts of interest.

## Data Availability

Source data for selected figures are provided with this paper. The remaining datasets used to support the findings of this study are available from the first author and corresponding authors upon request.
